# Chest associated to motor physiotherapy improves cardiovascular variables in newborns with respiratory distress syndrome

**DOI:** 10.1186/1755-7682-4-37

**Published:** 2011-10-26

**Authors:** Luiz Carlos de Abreu, Vitor E Valenti, Adriana G de Oliveira, Claudio Leone, Arnaldo AF Siqueira, Dafne Herreiro, Rubens Wajnsztejn, Katia V Manhabusque, Hugo Macedo Júnior, Carlos B de Mello Monteiro, Laís L Fernandes, Paulo HN Saldiva

**Affiliations:** 1 Departamento de Saúde Materno-infantil, Universidade de São Paulo, Av. Dr. Arnaldo, 715, São Paulo, SP. 01246-904, Brazil; 2 Departamento de Patologia, Faculdade de Medicina, Universidade de São Paulo (USP), Av. Dr. Arnaldo, 455, São Paulo, SP. 01246903, Brazil; 3 Laboratório de Escrita Científica. Faculdade de Medicina do ABC, Av. Príncipe de Gales, 821, Santo André, SP. 09060-650, Brazil; 4 Escola de Artes, Ciência e Humanidades da Universidade de São Paulo (USP), Rua Arlindo Béttio, 1000, São Paulo, SP. 03828-000, Brazil

## Abstract

**Background:**

We aimed to evaluate the effects of chest and motor physiotherapy treatment on hemodynamic variables in preterm newborns with respiratory distress syndrome.

**Methods:**

We evaluated heart rate (HR), respiratory rate (RR), systolic (SAP), mean (MAP) and diastolic arterial pressure (DAP), temperature and oxygen saturation (SO_2_%) in 44 newborns with respiratory distress syndrome. We compared all variables between before physiotherapy treatment vs. after the last physiotherapy treatment. Newborns were treated during 11 days. Variables were measured 2 minutes before and 5 minutes after each physiotherapy treatment. We applied paired Student t test to compare variables between the two periods.

**Results:**

HR (148.5 ± 8.5 bpm vs. 137.1 ± 6.8 bpm - p < 0.001), SAP (72.3 ± 11.3 mmHg vs. 63.6 ± 6.7 mmHg - p = 0.001) and MAP (57.5 ± 12 mmHg vs. 47.7 ± 5.8 mmHg - p = 0.001) were significantly reduced after 11 days of physiotherapy treatment compared to before the first session. There were no significant changes regarding RR, temperature, DAP and SO_2_%.

**Conclusions:**

Chest and motor physiotherapy improved cardiovascular parameters in respiratory distress syndrome newborns.

## Background

The respiratory distress syndrome (RDS) was reported in approximately 0.5% to 1% of newborns. The incidence and severity are directly related to prematurity degree. It affects around 50% of preterm newborns lighter than 1500 g and deaths associated to the disease; it usually occurs during acute phase of respiratory failure and is largely limited to extremely immature newborns, which birth weight is lower than 1000 g [1,2].

Neonatal physiotherapy is a procedure performed between clamping of umbilical cord and 28 days after delivery, which include newborn lung and motor handling [3]. Lung management aims to remove the excess of bronchial secretions. The adverse effect arising from excess secretions and the fact that their removal may significantly improve the specific conductance of the airways has been demonstrated in previous studies [4,5].

Physiotherapy procedures provides stability of hemodynamic variables, such as heart rate (HR) [6-9], the functional maintenance of newborn cerebral circulation and maintenance of airways with turbulent flow and minimal secretion, which allow a increased permeability and reduced number of intrinsic airway that contribute to increased airway resistance and decrease in gas changes physiological events [3].

There is controversy related to respiratory or chest physiotherapy in the neonatal period. Previous studies showed a reduction in hemodynamic variability of preterm infants and highlighted the beneficial therapeutic effects of interventional procedures of physiotherapy [3]. However, previous investigations reported deleterious effects, suggesting that the handling procedures of interventional therapy in preterm infants result in hemodynamic instability, and therefore it is not indicated [10].

Although the literature has already described the effects of chest physiotherapy on newborns with different lung diseases [11], no previous investigation analyzed chest associated to motor physiotherapy treatment on premature newborns with respiratory distress syndrome. Therefore, this study was undertaken to evaluate the effects of chest and motor physiotherapy treatment on hemodynamic variables in preterm newborns with respiratory distress syndrome.

## Methods

### Study Population

We evaluated 76 preterm newborns admitted in the Neonatal Intensive Care Unit with respiratory distress syndrome under noninvasive mechanical ventilation and treated with exogenous surfactant replacement (bovine type). Their parents or responsible signed a consent form. The protocol study was evaluated and approved by the Ethics Committee in Research of our University. Based on recommendations of the Ethics Committee in Research of our University, we did not use control group.

The concept of prematurity was adopted according to the World Health Organization, "preterm newborn younger than 37 gestational weeks (less than 259 days)". Gestational age was calculated based on reliable date of last menstrual period, data from the obstetric examination and ultrasound at the time of prenatal care. After birth we used the method of Capurro et al or method of Ballard [12,13] performed between the first 6 and 24 hours of life in order to determine gestational age. This procedure was carried out by the Neonatologist. Weight was expressed in grams, which was evaluated in the delivery room in all cases immediately after birth.

### Inclusion Criteria

We considered the following inclusion criteria: birth weight equal or higher than 1000 grams; clinical and radiological diagnosis of respiratory distress syndrome: clinical diagnosis was established when the newborn presented early respiratory distress (tachypnea, expiratory grunt, nasal flaring, chest retraction and cyanosis), early onset and progressive evolution. The radiological diagnosis was based on diffuse reticulogranular infiltrate (ground glass appearance), homogenously distributed in the lungs and presence of air bronchogram [14].

### Exclusion Criteria

We excluded 32 preterm newborns who presented congenital anomalies, genetic syndromes, neurological disorders, hydrops or congenital infection with clinical manifestations and death before the 3^rd ^day of life.

### Hemodynamic Variables

We evaluated the following variables: 1) respiratory rate (RR): measured in whole numbers from the number of breaths during 1 minute (cycles per minute - cpm); 2) Temperature: it was considered hypothermia axillary temperature below 309 K (36°C) in two consecutive measurements with an interval of at least two hours between measurements. We used a digital thermometer brand Microtherm^® ^for all newborns; Arterial pressure was measured by the oscillometric method, which uses an oscillometric monitor, oscillations are transmitted to the balloon located inside the pressure cuff and then captured by the device. The detection and quantification of the amplitude of these oscillations during the fall of automatic balloon pressure are transformed into blood pressure measurements by using various algorithms. Briefly, when the pressure is just above the pressure of the balloon, there was a rapid increase in the amplitude of oscillations it was considered as systolic arterial pressure (SAP). When there was a sharp drop in the amplitude of oscillations it was considered diastolic arterial pressure (DAP). Mean arterial pressure (MAP) was the lowest pressure balloon that still occurred in the oscillations of large amplitudes [15]. The device used to measure blood pressure was a noninvasive blood pressure monitor DIXTAL^® ^brand. The panel showed the values of SAP, DAP, MAP and HR. The variables HR (beats per minute - bpm) and SO_2_% (%) were measured and compiled by a multiparameter monitor Dixtal, model dx2020.

### Protocol Procedures

Newborns received physiotherapy treatment 6 hours after surfactant replacement, regardless of doses administration number. Procedures of neonatal physiotherapy were applied according to the following protocol: HR, RR, SO_2_%, SAP, MAP, DAP and body temperature measurement approximately 2 minutes before clinical sessions, chest and motor physiotherapy treatment and the same variables were measured again around 5 minutes after physiotherapy session. Each newborn were treated three times per day in an interval of 2 hours between each session, each treatment spent a maximum of 20 minutes (it was not included preparation, physical examinations, diagnostic imaging when performed and variables measurement according to the study protocol). The newborns were treated for 11 consecutive days.

### Statistical Analysis

In order to compare variables between the two periods (Before 1^st^treatment vs. After 11^st^treatment) we used paired Student t test. Differences were considered significant when the probability of a Type I error was less than 5% (p < 0.05).

## Results

Newborns birth weight ranged between 1000 and 2750 g; they did not receive supplemental oxygen before, during or after physiotherapy procedures. There was no significant difference regarding inspired oxygen fraction before and after treatment (before treatment - 0.6 ± 0.1 vs. after treatment - 0.3 ± 0.2; p = 0.111).

Most of the newborns received up to two doses of exogenous surfactant. Those who received great part of the surfactant weighed less than 1500 grams. Those with higher body weights received only one dose.

We compared all variables before and after physiotherapy treatment. HR was significantly (p < 0.001) reduced after the last physiotherapy treatment day compared to before the first session (Table 1). In relation to RR, we observed no significant difference between the last physiotherapy treatment compared to before the first session (Table 2).

**Table 1:** Mean, median, standard deviation, minimum and maximum values of HR (bpm) at rest measured on the first and last treatment day in newborns with RESPIRATORY DISTRESS SYNDROME

HR	Minimum	Median	Mean	Maximum	Standard Error
**Before 1^st ^treatment day**	126.0	147.0	148.5	165.0	8.5
After 11^th ^treatment day*	126.0	136.0	137.1	152.0	6.8

N = 44. *p < 0.001; Different of before first treatment day. HR: Heart rate.

**Table 2:** Mean, median, standard deviation, minimum and maximum values of RR (ipm) at rest measured on the first and last treatment day in newborns with RESPIRATORY DISTRESS SYNDROME

RR	Minimum	Median	Mean	Maximum	Standard Error
**Before 1^st ^treatment day**	22.0	47.0	47.5	64.0	6.4
**After 11^th ^treatment day**	32.0	40.0	42.4	70.0	8.9

N = 44. RR: Respiratory Rate.

With respect to arterial pressure, we noted that SAP and MAP (p = 001) reduced after the last session compared to before the first session, while there was no significant change regarding DAP (Table 3). We also evaluated oxygen saturation (Table 4) and temperature (Table 5) and it was not verified significant changes concerning these variables.

**Table 3:** Mean, median, standard deviation, minimum and maximum values of systolic, mean and diastolic arterial pressure (mmHg) at rest measured on the first and last treatment day in newborns with respiratory distress syndrome

Systolic Arterial Pressure	Minimum	Median	Mean	Maximum	Standard Error
**Before 1^st ^treatment day**	54.0	70.0	72.3	100.0	11.3

After 11^th ^treatment day*	52.0	63.5	63.6	80.0	6.7

**Mean Arterial Pressure**					

**Before 1^st ^treatment day**	34.0	56.0	57.5	83.0	12.0

After 11^th ^treatment day*	35.0	48.0	47.7	62.0	5.8

**Diastolic Arterial Pressure**					
**Before 1^st ^treatment day**	27.0	41.0	43.4	63.0	8.3
**After 11^th ^treatment day**	29.0	37.0	38.1	52.0	6.3

N = 44. *p = 0.001; Different of before first treatment day.

*p = 0.001

**Table 4:** Mean, median, standard deviation, minimum and maximum values of oxygen saturation (SO_2_%) at rest measured on the first and last treatment day in newborns with respiratory distress syndrome

SO_2_%	Minimum	Median	Mean	Maximum	Standard Error
**Before 1^st ^treatment day**	91.0	95.4	93.9	98.0	3.2
**After 11^th ^treatment day**	98.0	99.0	98.5	99.0	0.8

N = 44.

*p = 0.001

**Table 5:** Mean, median, standard deviation, minimum and maximum values of temperature (°C) at rest measured on the first and last treatment day in newborns with respiratory distress syndrome

Temperature	Minimum	Median	Mean	Maximum	Standard Error
**Before 1^st ^treatment day**	33.9	36.8	36.5	37.0	0.6
**After 11^th ^treatment day**	36.1	36.7	36.7	39.0	0.6

N = 44.

*p = 0.001

## Discussion

In this study we evaluated chronic effects of chest physiotherapy associated to motor physiotherapy on hemodynamic variables in respiratory distress syndrome to newborns. We found as main result that physiotherapy treatment reduced HR, SAP and MAP with no significant effect on RR, SO_2_% and temperature. Our investigation provides evidences that this physical resource contributes to reduce the hemodynamic instability in newborns with SDR.

At the end of physiotherapy treatment we reported significant reduction of HR, SAP and MAP in newborns with SDR. It supports the hypothesis that physiotherapy treatment improves newborns hemodynamic parameters, which diminishes cardiovascular instability and hence, decreases the likelihood of other systemic diseases development in newborns. Park et al [16] evidenced HR reduction during lung volume recruitment maneuvers onset in adult patients with respiratory SDR aged approximately 59 years old. The decrease in HR evidenced by them and reported in our study may be caused by augmented vagal tone and Valsalva maneuver like mechanisms [10]. However, there is difference between newborns and adults regarding their physiological responses [17].

We found that basal RR and SO_2_% were not changed after physiotherapy treatment. Conversely, Bernard-Narbonne et al [10] demonstrated that chest physiotherapy increased SO_2_% and tidal volume in children with acute bronchiolitis, which was linked to the improvement of bronchial sputum clearance. A previous investigation observed no improvement of lung function in children with exacerbated bronchial asthma who received chest physiotherapy [18]. The difference between those data may be explained by the type of disease and patients age. Other relevant factor that may be involved in this difference is the physiotherapy procedure, we used chest associated to motor physiotherapy. A variety of physiotherapy procedures is reported in the literature [19]. The most widely used and evaluated is the bronchial hygiene maneuver: chest (or percussion), vibration/vibrocompression maneuvers with an Ambu bag (bag-squeezing), aspiration and airway intubation, cough stimulation on posture and positioning of drainage and exercises respiratory liabilities in preterm infants [19].

In our procedures we associated chest physiotherapy to motor physiotherapy and we observed improvement of basal HR, SAP and MAP in premature newborns with SDR. Bronchial hygiene maneuvers are used to mobilize and remove secretions in airways in order to improve lung function. However, there are reports that these procedures may be malefic to preterm newborns [20]. There are investigations which do not indicate bronchial hygiene maneuvers for premature newborns weighting less than 1500 g in the first 3 days of life. They believed that it increases the possibility of cerebral bleeding [19]. Other reports suggest that bronchial hygiene maneuvers, especially clapping, may cause adverse effects in newborns, such as hypoxemia [20,21], ribs fracture and cerebral injuries [22]. In view of those researches, some studies tend to report clapping with adverse events in newborns [23]. It was demonstrated that the use of clapping is deleterious due the fragility and little size of newborns thorax, hence, it may increase the mechanical effects of clapping when compared to older subjects [23].

The results of our study suggest that chronic chest and motor physiotherapy helps to stabilize cardiovascular parameters in SDR newborns. Aspiration is a procedure often performed in order to keep airways permeability, especially in patients which do not cough regularly, as the newborn [24]. It is a procedure that requires extreme care in its implementation due to side effects that it may cause [25], due physiological changes induced by aspiration, such as hypoxemia and sympathetic hyperactivity [26-32] which may lead to peripheral vasoconstriction, increased blood pressure and arrhythmia, as well as changes in cerebral blood flow and elevated intracranial pressure [25]. Other effects are described, such as lesions of the tracheobronchial mucosa, bronchial perforation by suction catheter (with secondary pneumothorax), atelectasis (due to excessive negative pressure) and respiratory tract infections [24].

Along the physiotherapy treatment period we did not used Trendelenburg position, since it is not indicated in newborns because it may increase intracranial pressure and cause gastroesophageal reflux, which increases the risk of pneumonia [31].

We demonstrated that chronic physiotherapy maneuvers such as clapping or vibration followed by suction and postural drainage and/or vacuum improved cardiovascular variables in newborns with SDR. Physiotherapy treatment has received attention regarding preterm newborns with respiratory disorders, such as aspiration syndromes, respiratory distress syndrome, pneumonia, atelectasis and in those preterm newborns on mechanical ventilation. There are also indications of physiotherapy procedures in cases of airways secretion in newborns with negative prognostic [3]. Physiotherapy performed pre-and post-extubation showed improvement of pulmonary symptoms with reduced incidence of lung atelectasis after extubation [3]. Physiotherapy results in lung mechanical effects, providing optimal respiratory function in order to facilitate gas exchange and adjust ventilation-perfusion adequacy of respiratory support, to prevent and treat pulmonary complications, to provide good maintenance of airways and to facilitate weaning from mechanical ventilation and oxygen therapy [33].

There is not enough evidence to determine whether active chest physiotherapy is of benefit to neonates on mechanical ventilation. Babies who require mechanical ventilation are at risk of lung collapse from increased secretions. Chest physiotherapy (patting or vibrating the chest) is used to improve clearance of secretions from the airway to try to prevent lung collapse. This review found no clear overall benefit or harm from chest physiotherapy. Some individual chest physiotherapy techniques were more beneficial than others in resolving atelectasis and maintaining oxygenation. These results do not support one technique over another. Due to the limited number, poor quality and age of trials in this review, there is not enough evidence to determine whether or not chest physiotherapy is beneficial or harmful in the treatment of infants being ventilated in today's intensive care units. Further good quality trials are needed to address this issue [33].

However, chest and motor physiotherapy treatment were able to improve basal cardiovascular parameters in newborns with respiratory distress syndrome newborns. Therefore, we indicate performing chest and motor physiotherapy in SDR newborns.

## Competing interests

The authors declare that they have no competing interests.

## Authors' contributions

All authors participated in the acquisition of data and revision of the manuscript. All authors determined the design, interpreted the data and drafted the manuscript. All authors read and gave final approval for the version submitted for publication.
